# The effect of IPC on central and peripheral fatiguing mechanisms in humans following maximal single limb isokinetic exercise

**DOI:** 10.14814/phy2.14063

**Published:** 2019-04-25

**Authors:** Samuel L. Halley, Paul Marshall, Jason C. Siegler

**Affiliations:** ^1^ Sport and Exercise Science School of Science and Health Western Sydney University Sydney New South Wales Australia

**Keywords:** Blood occlusion, central fatigue, exercise, peripheral fatigue

## Abstract

Ischemic preconditioning (IPC) has been suggested to preserve neural drive during fatiguing dynamic exercise, however, it remains unclear as to whether this may be the consequence of IPC‐enhanced muscle oxygenation. We hypothesized that the IPC‐enhanced muscle oxygenation during a dynamic exercise task would subsequently attenuate exercise‐induced reductions in voluntary activation. Ten resistance trained males completed three 3 min maximal all‐out tests (AOTs) via 135 isokinetic leg extensions preceded by treatments of IPC (3 × 5 min bilateral leg occlusions at 220 mmHg), SHAM (3 × 5 min at 20 mmHg) or CON (30 min passive rest). Femoral nerve stimulation was utilized to assess voluntary activation and potentiated twitch torque during maximal voluntary contractions (MVCs) performed at baseline (BL), prior to the AOT (Pre), and then 10 sec post (Post). Tissue oxygenation (via near‐infrared spectroscopy) and sEMG activity was measured throughout the AOT. MVC and twitch torque levels declined (MVC: −87 ± 23 Nm, 95% CI = −67 to −107 Nm; *P* < 0.001, twitch: −30 ± 13 Nm; 95% CI = −25 to −35 Nm; *P* < 0.001) between Pre and Post without reductions in voluntary activation (*P* = 0.72); there were no differences between conditions (MVC:* P* = 0.75, twitch: *P* = 0.55). There were no differences in tissue saturation index (*P* = 0.27), deoxyhemoglobin concentrations (*P* = 0.86) or sEMG activity (*P* = 0.92) throughout the AOT. These findings demonstrate that IPC does not preserve neural drive during an all‐out 3 min isokinetic leg extension task.

## Introduction

Ischemic preconditioning (IPC) was initially developed as a protective technique against myocardial ischemia/reperfusion injuries (Murry et al. 1986). It has recently been introduced in sporting contexts as an ergogenic aid due to the similar metabolic perturbation profile between high intensity exercise and mild ischemic insults (de Groot et al. 2010; Marongiu and Crisafulli 2014; Quindry 2017; Quindry and Hamilton 2013). The IPC technique generally imposes 3–4 × 5 min cycles of contralateral limb occlusion followed by a 5 min reperfusion period, accumulating 30–40 min of sustained limb ischemia (Horiuchi 2017). Performance trials have demonstrated small albeit inconsistent ergogenic effects, ranging across modalities (running, rowing, cycling, swimming), time domains (<1–30 min) and intensities (submaximal, sustained high intensity and all‐out effort) (Bailey et al., 2012b; Patterson et al. 2015; Lindsay et al. 2017; Jean‐St‐Michel et al. 2011; Ferreira et al. 2016; Lisboa et al. 2017) (Kjeld et al. 2014). These performance effects have often been attributed to a combination of peripheral vasodilation and increases in mitochondrial efficiency (Andreas et al. 2011; Semenza 2011; Horiuchi et al. 2015; Paradis‐Deschênes et al. 2016, 2017; Tanaka et al. 2016; Lindsay et al. 2017). However, a growing body of research has suggested that IPC may also augment neural mechanisms concomitant to the preservation of skeletal muscle function (Cruz et al. 2015, 2016; Hyngstrom et al. 2018).

Recent clinical research has demonstrated acute improvements in maximal strength corresponding with increases in surface electromyography (sEMG) signal amplitude after IPC (Hyngstrom et al. 2018). Similarly, enhanced sEMG signals have been seen in both aerobic and sprint cycling, where increased sEMG to power output ratios have suggested that IPC increases skeletal muscle activation (Cruz et al. 2015, 2016). Based on these findings, it was suggested that IPC facilitated increased muscle activity via a disruption to the central feedback loop (Cruz et al. 2017). This disruption is proposed to arise through inhibited signaling from metabolically sensitive group III and IV afferent sensory fibers as a consequence of the metabolic autacoids produced during the IPC cycles (Downey et al. 2007; Redington et al. 2012; Amann et al. 2013; Blain et al. 2016; Burns et al. 2016; Cruz et al. 2017). However, whilst sEMG can provide an estimate of gross nervous system output, the multitude of unrelated factors contributing to the signal amplitude confounds any mechanistic insight as to where within the neural circuitry IPC may be conferring its effect (Farina et al. 2010). In light of this, we recently used the interpolated twitch technique during a maximal, 2‐min isometric leg extension task to better clarify whether IPC influences centrally mediated factors (e.g. neural drive) associated with maintaining task performance (Halley et al. 2018). Contrary to previous reports (Cruz et al. 2015, 2016), we did not observe an effect of IPC on sEMG amplitude nor did we see any improvement in the additional measures of neural drive (e.g. voluntary activation) (Halley et al. 2018).

A notable point of difference between these studies, however, was our use of a static, prolonged maximal voluntary contraction (Halley et al. 2018) while the cycling tasks were more dynamic in nature (Cruz et al. 2015, 2016). Although we hypothesized the sustained maximal effort would provide a favorable environment for IPC to influence neural drive (Taylor et al. 2000; Martin et al. 2006), the isometric task may have restricted skeletal muscle oxygen delivery through augmented intramuscular pressures (McNeil et al. 2015). Moreover, the increased neural activity observed in both cycling studies was associated with IPC‐mediated improvements in aerobic energy provision (as inferred via augmented hemoglobin kinetics) and therefore endurance capacity (Cruz et al. 2015, 2016). More recently, Griffin and colleagues have also demonstrated significant improvements in the ability to sustain maximal rates of aerobic metabolism (e.g. critical power) after IPC, but conversely, did not observe a differential effect on skeletal muscle oxygenation (Griffin et al. 2018). As these authors did not measure neural activity, we cannot discern whether IPC‐mediated preservation of neural signaling compensated for the negligible change in aerobic energy provision.

Therefore, the aim of this study was to determine whether IPC, coupled with a dynamic exercise task designed to optimize oxygen delivery to skeletal muscle, augments neural drive during fatiguing exercising. Specifically, we examined whether a standard bout of IPC (3 × 5 min cycles) better preserves muscle activity during, and voluntary activation following a 3‐min maximal power test comprised of rhythmic dynamic single‐leg extensions. Given recent evidence (Cruz et al. 2015, 2016; Tanaka et al. 2016), it was hypothesized that IPC would improve performance of the test through an attenuation of centrally mediated declines in neural drive (e.g. voluntary activation) induced by the fatiguing protocol.

## Methods

### Ethical approval

All procedures in this study were approved by the Western Sydney University Human Research Ethics Committee (approval code H12114) and were conducted in accordance with the Declaration of Helsinki, except for registration in a database. Ten resistance trained men with a minimum training history of at least two resistance training sessions per week for the previous 6 months (mean ± SD) (age: 24.1 ± 3, weight: 80.5 ± 7 kg) volunteered to participate in this study after providing informed written consent. Minimum sample size was calculated from an a priori power analysis employing the small effect size threshold value of *d* = 0.2 as the indication of the smallest worthwhile change using a customized spreadsheet (Hopkins 2006).

### Experimental design

**Figure 1:**
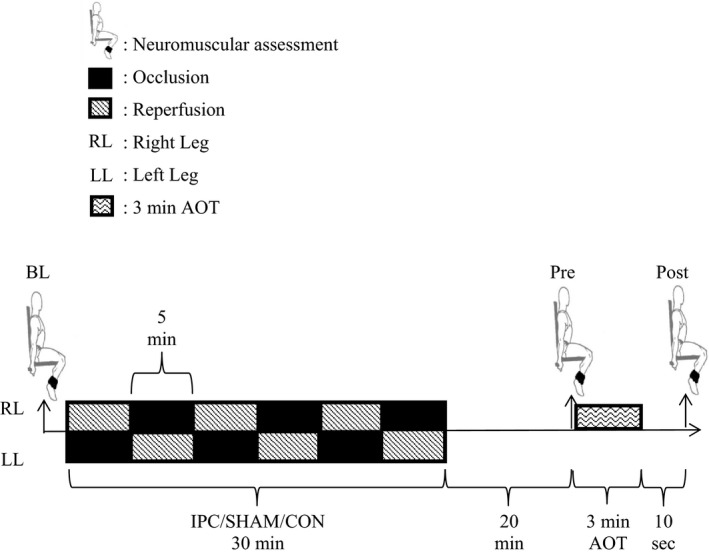
Study flow diagram.

A study flow diagram is provided in Figure 1. During a preliminary session, participants were familiarized with all procedures involved in the study and provided with pretest guidelines in regards to restriction of caffeine (12 h), alcohol (24 h), strenuous lower body exercise (48 h), and instructions as to replication of caloric and macro intake (24 h) prior to the experimental trials. The preliminary session included exposure to the femoral nerve stimulation protocols during a 3–5 sec maximal voluntary isometric knee extension contraction (MVC), the fatiguing protocol (a 3‐min all out maximal power dynamic single‐leg extension test (AOT)) and the IPC protocol. All testing was performed in a climate controlled laboratory (mean ± SD) (temperature: 23 ± 1°C; humidity: 50 ± 3%), at the same time of day and on the right leg over 3 days in a single blind, repeated measures and randomized fashion with 48 h between each session.

Upon arrival to the laboratory, participants were fitted for sEMG (see Surface EMG) and nerve stimulation (see Femoral Nerve Stimulation) electrodes as well as a near infrared spectrometer (NIRS) (moorVMS‐NIRS; Moor Instruments Ltd, Axminster, Devon, UK). Neuromuscular function (see Neuromuscular Function) was then assessed at baseline prior to the intervention (BL), 20 min after the intervention (e.g. IPC or sham) and 1 min prior to the AOT (Pre), and lastly 10 sec after completing the AOT (Post). All participants performed the AOT under three separate intervention protocols; a control (CON), a sham occlusion (SHAM) or the IPC protocol (IPC). The interventions were performed with the participant lying in a supine position, and blood flow to the lower leg restricted via the inflation of a blood pressure cuff to either 220 mmHg (IPC) or 20 mmHg (SHAM) for three 5 min periods on each leg, each interspersed by a 5 min reperfusion (Salvador et al. 2016). Occlusion alternated between contralateral limbs ensuring a total IPC or SHAM time of 30 min whilst the CON protocol consisted of 30 min of passive rest. A plateau in the NIRS‐derived deoxhemoglobin concentration signal was observed in every participant at the end of each 5‐min cycle, and was taken as a sign of occlusion. The inflatable cuff (Hokanson CC17, Bellevue, WA, US) was positioned on the upper leg where the adductor longus muscle attaches to the inguinal ligament and pressurized via an automated system (Hokanson E20/AG101Rapid Cuff Inflation System, Bellevue, WA, USA). The cuff was 18 cm wide with an adjustable length of 108 cm. To minimize any placebo effect, participants were told that the purpose of the study was to compare the impact of two different cuff pressures that could both alter performance (Paradis‐Deschênes et al. 2016). The participant then completed an additional 20 min rest prior to the onset of the AOT (Salvador et al. 2016).

After the 20 min rest, the AOT was performed on a dynamometer (Biodex Pro 4, Biodex Medical Systems, Inc., Shirley, New York, USA) as a 3‐min all out maximal power test. The task was comprised of 135 repetitions of an isokinetic (300°/sec extension: 500°/sec flexion), knee extension movement with the range of motion set to 75° (from the start position, 90°knee flexion) and performed at a frequency of 45 repetitions per minute (Griffin et al. 2018). Each contraction was performed maximally with no pacing to ensure that a plateau in power output was achieved prior to the conclusion of the test. No knowledge of work rates or timing was given to the participants during the test. Coefficient of variation (CV's) of the performance outcomes of the AOT were between 7% and 16%, respectively.

### Neuromuscular function

Maximal voluntary torque and measures of voluntary and muscle activation were assessed using the same dynamometer (Biodex Pro 4). Participants were positioned upright in the dynamometer with thigh/trunk and knee angles of 90°, and the centre of rotation of the lever arm aligned with the sagittal plane axis of the knee joint. Participants were secured with an adjustable thigh strap and contralateral diagonal shoulder straps securing the trunk. A padded ankle cuff was placed superior (3 cm) to the lateral malleolus. Prior to performing exercise, participants completed a standardized warm up of six submaximal, 2–3 sec isometric contractions (2 × 25%, 2 × 50%, and 2 × 75% of perceived maximal effort). Torque output signals were directly sampled from the dynamometer at 1000 Hz (Powerlab, ADI Instruments, Sydney, NSW, Australia) and low pass filtered at 10 Hz. Torque signals were calibrated for each participant's limb weight (following positional securing via straps), and a predetermined calibration factor was applied for conversion of the recorded voltage to units of torque (Nm). For baseline measures four MVCs were performed; two with supramaximal nerve stimulation (*see* Femoral Nerve Stimulation) and two without, with one minute rest between each effort. The highest of the four MVCs was used as the BL value. Thereafter, only 1 MVC was performed pre and post the exercise task (e.g. AOT). Each MVC was instructed to be performed as hard and as fast as possible and maintained for 3–4 sec. Strong verbal encouragement was provided during all MVCs. The maximal torque measure recorded for each MVC was taken as the highest torque value achieved during the contraction prior to the nerve stimulation. CVs of 5% and 9% were recorded for MVCs and rates of torque development (RTD) between trials, similar to previously published CV values (Place et al. 2007; Buckthorpe et al. 2012).

### Femoral nerve stimulation

A 4 × 3 cm aluminium foil strip coated with Conductive Adhesive Hydrogel (Ten20; Weaver and Company, Aurora, CO) was placed on the lateral aspect of the hip, inferior to the iliac crest, as the anode. To identify nerve location for cathodal stimulation of the femoral nerve (Digitimer model DS7AH stimulator, Welwyn Garden City, UK), a rubber‐insulated portable cathodal probe was used. The probe was moved around the femoral triangle using a stimulus intensity of 30 mA until the largest muscle compound action potential (M‐wave) was elicited from both the VL and VM recording sites. For all stimulations, a 200 *μ*sec square‐wave pulse was applied at 400 V, with each stimulation being a doublet (two pulses applied to the femoral nerve with a 100 msec inter stimulus interval; 10 Hz stimulation). When the optimal nerve location was identified it was marked with a felt‐tip pen and a 20 mm diameter surface electrode was applied (Kendall 700; Tyco Healthcare Group, Mansfield, MA). Thereafter, stimulus intensity was increased in 10 mA increments with 20 sec rests between stimuli until plateaus in VL and VM M‐wave amplitudes (Mmax) and the size of the knee‐extensor twitch torque were observed. The maximal stimulus intensity (in milliamperes) was recorded and multiplied by 130% to establish the test intensity (range for participants 70–290 mA; VL Mmax 13.1 ± 6.8 mV and VM Mmax 12.1 ± 3.7 mV). Twitch torque CVs of 2.2% were recorded across trials, similar to that of previous reliability studies (Place et al. 2007).

### Surface EMG (sEMG)

Knee extensor sEMG was recorded during the experimental trials with Ag–AgCl surface electrodes (Maxsensor; Medimax Global, Seven Hills, NSW, Australia) positioned over the vastus medialis (VM) and vastus lateralis (VL) on the right thigh. Electrodes were placed in the centre of the muscle belly with the inferior VL and VM electrodes positioned 8–12 cm and 3–4 cm from the lateral aspect of patella, respectively. A self‐adhesive reference electrode was attached to the patella on the left leg. All placement sites were shaved, exfoliated and cleaned with an alcohol swab, while electrodes and cables were also taped to avoid any movement artefact. Electrode placement was recorded to ensure consistency within and between testing sessions. Raw electrode output was preamplified and bandpass filtered, with a bandwidth frequency ranging from 20 to 500 Hz (common mode rejection ratio > 90 dB; impedance input = 100 MΩ; gain = 1000). Peak and maximal rate of amplitude development were quantified for both VM and VL. Processing of all neuromuscular data was performed using MATLAB software (R2010a; The MathWorks Inc., Natick, MA).

### Near‐infrared spectroscopy (NIRS)

Oxygenation status of skeletal tissue was assessed using a spatially resolved, dual wavelength NIRS apparatus (moorVMS‐NIRS; Moor Instruments Ltd, Axminster, Devon, UK). NIRS data were collected for tissue saturation index (TSI) as a percentage of haemoglobin oxygen saturation (%) and deoxyhemoglobin concentrations (HHb) in arbitrary units (AU). After cleaning the skin with an alcohol swab, the NIRS device was fixed directly above the belly of the rectus femoris muscle at the mid‐point between the inguinal fold and the top of the patella on the most anterior aspect. The device was fitted with an adhesive black silicone mould to reduce movement artefact and eliminate potentially interfering background noise. Skinfold thickness was measured at the site of the application of the NIRS $\left(\overset{¯}{x}=12.8\;\mathrm{mm}\right)$ using a Harpenden skinfold calliper (British Indicator Ltd., Burgess Hill, West Sussex*,* UK) during the familiarization session, and was less than half the distance between the emitter and the detector (i.e. 30 mm). This thickness is adequate to let near infrared light through muscle tissue, and coupled with the anatomical location of the NIRS, minimizes measurement variation. The position on the participant was noted and replicated in the following trials. The pressure cuff was positioned above the NIRS device, which did not affect the placement of the device during occlusions. A modified form of the Beer‐Lambert law, using two continuous wavelengths (760 and 850 nm) and a differential optical path length factor of 4.95, was used to calculate micro molar changes in tissue hemoglobin concentrations. NIRS data were acquired at 5 Hz. At rest, once positioned within the dynamometer, the signal was stabilized and 1 min of baseline values was analyzed pre intervention. The NIRS signals were recorded throughout the experimental trials with average values reported at baseline, during the intervention and throughout the AOT. Peak values were also reported during the intervention and throughout the AOT. The times to peak HHb concentrations, measured from the start of the AOT, were also reported.

### Data processing

During all MVCs, the following torque variables were recorded: (i) the maximal voluntary torque (MVT; in Nm), calculated as the highest torque value throughout the entire MVC; and (ii) the maximal rate of torque development (RTDmax; in Nm·sec^−1^), calculated as the greatest average 10 msec slope of the torque–time curve during the initial 100 msec of the contraction.

Voluntary activation (VA) was estimated using the superimposed twitch technique (Merton 1954) according to the following formula (Strojnik and Komi 1998): VA (%) = 100 − [*D × *(*T*sup/MVT)/RT] × 100, where *D* is the difference between the torque level immediately before the superimposed twitch (*T*sup) and the maximal torque recorded during the twitch, MVT is maximal voluntary torque during the entire contraction (not including the twitch response), and RT is the maximal amplitude of the resting potentiated twitch. The following variables were calculated from the resting potentiated twitch: (i) RT amplitude (in newton metres (Nm)); (ii) time to peak torque production derived from the RT (TTP; in milliseconds (msec)); (iii) the half‐relaxation time (½ RT; in msec), which was defined as the time elapsed from the peak twitch torque to 50% peak twitch torque.

The following variables were processed from each MVC for VL and VM sEMG signals: (i) maximal sEMG amplitude of VL (VLmax) and VM (VMmax), processed as the greatest average root mean square over a 250 msec period from the beginning of the contraction to prior to the supramaximal stimulation; and (ii) maximal rate of sEMG rise (VLRERmax and VMRERmax), calculated from the greatest average 10 msec slope throughout the initial 100 msec of the contraction. All sEMG signals were normalized to their respective M‐wave.

From the AOT the following performance variables were recorded: (i) end test power (ETP); calculated as the average peak power output from the repetitions performed in the last 30 sec of the test, (ii) work above end test power (W^ETP); calculated as the sum of work completed above ETP, (iii) peak power output (PPO); calculated as the highest five point running average of power output and (iv) total work done (TWD). Peak VL and VM signals were also collected for each repetition of the AOT and analyzed as an average signal amplitude during the ETP, W^ETP, and PPO periods of the test. All sEMG signals were normalized to their respective M‐waves.

### Statistical analyses

Descriptive data are presented as mean ± SD. All statistical analyses were completed using IBM SPSS Statistics version 25 (SPSS Inc., Chicago, IL). The Shapiro–Wilk test was applied to assess normality of distribution. ETP, W^ETP, PPO, TWD, and NIRS data were analyzed for differences using a one‐way ANOVA for repeated measures. BL, Pre, and Post neuromuscular assessment data (MVT, RTDmax, VA, VLmax and VMmax, VLRERmax, and VMRERmax and RT) were analyzed using a two‐way (condition × time) ANOVA for repeated measures. In the event of a significant *F* ratio, *post hoc* comparisons were made using a Bonferroni correction. Two‐tailed statistical significance was accepted at *P* < 0.05. Mean differences with standard deviations (SD) were calculated when significant changes over time or when differences between conditions were observed.

## Results

### Maximal torque production

Maximal torque output was not different between BL (252 ± 46 Nm) and Pre (238 ± 44 Nm), but decreased at Post (151 ± 28 Nm) following the AOT (mean decline of 87 ± 23 Nm, 95% CI = −67 to −107 Nm; *P* < 0.001). There were no differences between conditions (*P* = 0.75). Similarly, RTDmax did not change between BL (1709 ± 328 Nm·sec^−1^) and Pre (1575 ± 376 Nm·sec^−1^) but decreased at Post (mean declines of 572 ± 124 Nm·sec^−1^, 95% CI = −353 to −792 Nm·sec^−1^; *P* < 0.001). There were no differences between conditions (*P* = 0.70).

### Descending central drive

Voluntary activation was unchanged throughout the experimental trials (BL: 87 ± 7%, Pre: 85 ± 8%, Post: 88 ± 5%; *P* = 0.72). There were no changes in peak sEMG amplitude or RERmax throughout the trials or between conditions for VL (Table 1). For VM, there was a main effect of time for peak sEMG amplitude (*P* = 0.05), however, post hoc analysis revealed no pairwise differences (Table 1). There were no differences between conditions in VM or VL sEMG measures in any variable throughout the AOT (Table 2.)

**Table 1:** Data are presented as mean ± SD

	VM						VL					
	Max (%)			RERmax (%)			Max (%)			RERmax (%)		
	CON	SHAM	IPC	CON	SHAM	IPC	CON	SHAM	IPC	CON	SHAM	IPC
BL	15 ± 4	16 ± 8	15 ± 6	763 ± 343	732 ± 388	850 ± 585	11 ± 5	11 ± 5	12 ± 6	567 ± 295	585 ± 300	396 ± 169
PRE	12 ± 4	15 ± 4	16 ± 5	653 ± 345	524 ± 341	844 ± 604	12 ± 6	10 ± 5	13 ± 6	585 ± 384	365 ± 142	469 ± 193
POST	12 ± 3	13 ± 4	14 ± 5	656 ± 444	815 ± 722	756 ± 401	10 ± 4	10 ± 5	12 ± 7	530 ± 349	424 ± 178	638 ± 335

The sEMG data for VM (VMmax, VMRERmax) and VL (VLmax, VLRERmax), (all expressed as percentages of respective M‐waves) for each MVC performed at baseline (BL), prior to the 3‐min AOT (Pre) and post‐3 min AOT (Post) for control (CON) (*n* = 10), sham (SHAM) (*n* = 10) and IPC (*n* = 10). Abbreviations: sEMG, surface electromyograms normalized to M‐waves; VL, vastus lateralis; VM, vastus medialis; RERmax, maximum rate of sEMG rise.

**Table 2:** Data are presented as mean ± SD

	VM (%)			VL (%)		
	CON	SHAM	IPC	CON	SHAM	IPC
PPO	14 ± 15	10 ± 2	13 ± 7	9 ± 2	9 ± 3	8 ± 3
W^ETP	15 ± 14	10 ± 2	12 ± 6	8 ± 2	8 ± 3	8 ± 3
ETP	13 ± 11	9 ± 2	10 ± 5	7 ± 2	7 ± 2	7 ± 3

The sEMG data for VM and VL during the 3 min all out test (AOT), (all expressed as percentages of respective M‐waves) averaged across during the time window of peak power output (PPO), power output above the plateau reached at the end of the test (W^ETP) and the plateau reached at the end of the test (ETP) for control (CON) (*n* = 10), sham (SHAM) (*n* = 10) and IPC (*n* = 10). Abbreviations: sEMG, surface electromyograms normalized to M‐waves; VL, vastus lateralis; VM, vastus medialis.

### Resting twitch characteristics

The resting twitch torque declined from Pre after the AOT (mean decline of 30 ± 13 Nm; 95% CI = −25 to −35 Nm; *P* < 0.001; Table 3). The ½ RT (mean increase in 33 ± 26 msec; 95% CI = 14–51 msec; *P* < 0.01; Table 3) and TTP (mean increase in 16 ± 13 msec; 95% CI = 6–26 msec; *P* < 0.01; Table 3) both increased after the AOT. There were no differences between conditions in any resting twitch characteristics.

**Table 3:** Data are presented as mean ± SD

	RT (Nm)			TTP (msec)			½ RT (msec)		
	CON	SHAM	IPC	CON	SHAM	IPC	CON	SHAM	IPC
BL	94 ± 17	92 ± 17	95 ± 15	103 ± 8	95 ± 7	103 ± 8	100 ± 25	106 ± 6	111 ± 30
PRE	90 ± 15	91 ± 14	91 ± 13	99 ± 7	101 ± 9	100 ± 10	103 ± 9	102 ± 10	100 ± 9
POST	61 ± 9*	62 ± 10*	62 ± 6*	121 ± 16*	120 ± 12*	114 ± 19*	129 ± 27*	135 ± 32*	138 ± 25*

Data for amplitude of the peak resting twitch force (RT), the time‐to‐peak twitch (TTP), the half‐relaxation time (½ RT) after maximal voluntary contractions at baseline (BL), prior to the 3‐min AOT (Pre) and post 3‐min AOT (Post) for control (CON) (*n* = 10), sham (SHAM) (*n* = 10) and IPC (*n* = 10).

* Significantly different from BL (*P* < 0.05).

### Oxygenation

At BL there were no differences between conditions in TSI (CON: 68.7 ± 5.1%, SHAM: 67.5 ± 4.7%, IPC: 67.7 ± 5.9%) or HHb (CON: 96 ± 31 AU, SHAM: 104 ± 36 AU, IPC: 113 ± 41 AU). During the intervention, in the IPC condition peak HHb was elevated versus CON but not SHAM (mean increase vs. CON of 99 ± 95 AU; 95% CI = 4 to 195 AU; *P* < 0.05, vs. SHAM 85 ± 92 AU; *P* = 0.07; Table 4) and average TSI was lower versus both CON and SHAM (mean decrease vs. CON of 10.6 ± 5.9; 95% CI = −5 to −16%; *P* < 0.001, vs. SHAM 8.0 ± 6.3%; 95% CI = −2 to −14%; *P* < 0.05; Table 4). During the AOT, there were no differences between conditions for either W^ETP or ETP in average or minimal tissue saturation (TSI) or maximal or average deoxyhemoglobin (HHb). There was no difference between conditions in the time to peak HHb concentrations.

**Table 4:** Data are presented as mean ± SD

Average	TSI (%)			HHb (AU)		
	CON	SHAM	IPC	CON	SHAM	IPC
Intervention	76.0 ± 3.0	73.3 ± 5.7	65.4 ± 6.1*	67 ± 17	71 ± 28	100 ± 44
W^ETP	55.5 ± 11.1	58.9 ± 10.4	58.2 ± 8.7	139 ± 71	137 ± 71	140 ± 75
ETP	59.3 ± 10.0	61.8 ± 9.5	60.4 ± 8.6	152 ± 82	134 ± 69	140 ± 67
End	59.2 ± 9.9	61.3 ± 9.7	60.1 ± 8.7	152 ± 81	134 ± 68	140 ± 67
Peak	CON	SHAM	IPC	CON	SHAM	IPC
Intervention	74.2 ± 3.7	70.2 ± 3.8	40.9 ± 13.2*	73 ± 19	86 ± 33	171 ± 102*
W^ETP	52.0 ± 14.3	54.6 ± 13.9	54.6 ± 12.3	175 ± 97	157 ± 80	162 ± 87
Time to Peak (s)	24.7 ± 7.0	29.6 ± 6.7	22.1 ± 7.8	37 ± 13	30 ± 10	39 ± 15

Average and peak data for tissue saturation index (TSI) and deoxyhemoglobin (HHb) during the intervention, the window of power output above the plateau reached at the end of the test (W^ETP), the plateau reached at the end of the test (ETP) and the last 10s of the test (End) for control (CON) (*n* = 10), sham (SHAM) (*n* = 10) and IPC (*n* = 10). Time to peak represents the time to minimum TSI and maximum HHb levels.

* Significantly different from CON and SHAM (*P* < 0.05).

### Performance

There were no differences between conditions in ETP, Ave Power W^ETP, W^ETP, TWD, or PPO (Table 5).

**Table 5:** Data are presented as means ± SD

	AOT Physical Characteristics		
	CON	SHAM	IPC
ETP (W)	260 ± 66	271 ± 78	251 ± 64
W^ETP (J)	2997 ± 978	3051 ± 1158	3158 ± 917
TWD (J)	8817 ± 2089	9211 ± 2020	8770 ± 2050
PPO (W)	742 ± 127	744 ± 114	750 ± 126

The performance data for the 3 min all out test (AOT) with the end test power (ETP), work done above the rate of end test power (W^ETP), total work done (TWD) and peak power output (PPO) for control (CON) (*n* = 10), sham (SHAM) (*n* = 10), and IPC (*n* = 10).

## Discussion

The primary aim of this study was to determine whether IPC would attenuate the decline in neural drive following a dynamic, 3‐min leg extension protocol designed to optimize skeletal muscle oxygen delivery. Contrary to our hypothesis, IPC did not affect voluntary activation nor did it influence other measures of neural activity (e.g. sEMG amplitude) or peripheral fatigue characteristics. Furthermore, IPC did not attenuate the decline in functional measures of performance (MVC, RTD, PPO, etc.), nor did it alter tissue oxygenation during the 3‐min exercise.

In contrast to other reports (Griffin et al. 2018), IPC had a nominal effect on neuromuscular fatigue following the maximal effort knee extension task (e.g. AOT). Our hypothesis was based on previous studies theorizing that IPC may preserve skeletal muscle activation throughout exercise as a result of an overshoot in central motor drive induced via a disruption to the central feedback loop mechanism (Amann et al. 2011; Crisafulli et al. 2011; Blain et al. 2016; Cruz et al. 2017). This disruption is thought to occur through an inhibition of afferent signaling from metabolically sensitive group III and IV fibres resulting from the desensitization of the muscle afferents due to over stimulation during the IPC procedure (Burns et al. 2016) and/or a reduced excitability of the muscle afferents due to opioid receptor activation by circulating endogenous opioids released during the IPC stimulus (Downey et al. 2007; Amann et al. 2009). Regardless of mechanistic pathway, any influence IPC might have on these sensory fibers should manifest in changes to measures of central drive (e.g. voluntary activation) (Babault et al. 2006; Place et al. 2007; Amann et al. 2009). However, and consistent with our previous observations, IPC did not affect any measures of central fatigue used in this study (Table 3). Collectively, this raises the possibility that the increased sEMG activity observed in previous studies (Cruz et al. 2015, 2016; Hyngstrom et al. 2018) may be unrelated to central factors associated with neural drive. Alternatively, the increased sEMG signals reported in previous studies may be a result of the lack of control for contraction velocity. Contraction velocity is known to confound sEMG measures because of the effect dynamic contractions have on the stability of the skin‐muscle interaction and therefore the action potentials recorded by the surface electrodes (Luca 1997). Despite prior reports of IPC improving contraction and relaxation times, contraction velocity has not been controlled in previous studies and therefore is an unaccounted variable for explaining the increased sEMG activity (Barbosa et al. 2014). However, an isokinetic dynamometer was used to control and account for the contraction velocities in this study with no observable differences in sEMG amplitudes (Table 2). Consequently, it may be plausible that previous observations of increased sEMG activity were reflective of variations in contraction velocities rather than enhanced efferent drive.

A dynamic fatiguing protocol was used in this study to ensure adequate muscle perfusion during exercise (Bailey et al., 2012a; Paradis‐Deschênes et al. 2016; Tanaka et al. 2016; Halley et al. 2018), and theoretically allow the IPC to promote the acute upregulation of vasodilatory factors such as adenosine and nitric oxide (Horimoto et al. 2000; Kontos 2001; Kimura et al. 2007) and preserve muscle function similar to previous reports (Cruz et al. 2015, 2016). However, there was no notable preservation of muscle function throughout (Table 5) or following (Table 3) the dynamic protocol. Additionally, and in contrast to our previous findings under isometric conditions (Halley et al. 2018), IPC did not increase hemoglobin volume or accelerate oxygen dissociation (Table 4). We believe this disparate outcome in tissue saturation kinetics may be related to the severity of peripheral fatigue induced by the isometric protocol. Despite the shorter duration of the isometric protocol (2 vs. 3 min) (Halley et al. 2018), declines in muscle contractility were far greater than in the dynamic leg extension protocol (RT ~ 32% lower and ½ RT ~ 33% longer) (Halley et al. 2018). The task demand (e.g. 100% MVC), coupled with the restricted muscle perfusion, may also explain the increase in localized O_2_ delivery after IPC (~35%) observed only in the 2‐min isometric protocol (Halley et al. 2018). Interestingly, others reporting comparable humoral effects after IPC have observed performance improvements during lower intensity efforts (e.g. 20% MVC to task failure), albeit without a sham condition (Tanaka et al. 2016). The substantial difference in contraction intensity between these studies (20% vs. 100% MVC (Halley et al. 2018; Kraus et al. 2015)) may further suggest that the ergogenic effects of IPC are dependent on a combination of task demand and the extent of localized fatigue in the muscle (Marshall et al. 2015).

**Figure 2:**
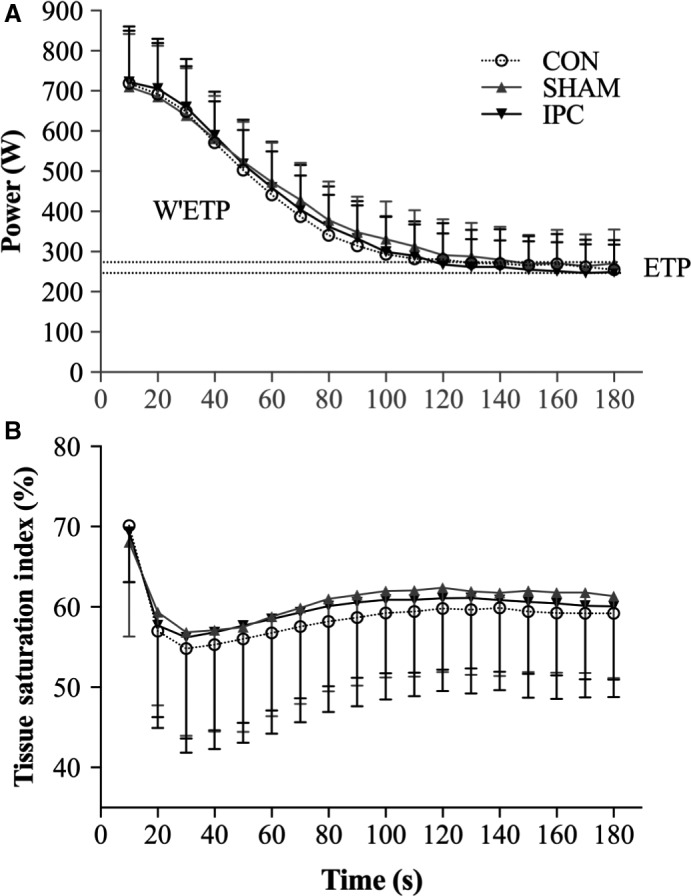
Data are presented as means ± SD. (A) Average power production at 10 sec increments throughout the AOT for control (CON) (*n* = 10), sham (SHAM) (*n* = 10) and IPC (*n* = 10). (B) Average tissue saturation index at 10s increments throughout the AOT for CON, SHAM and IPC. Abbreviations: the end test power (ETP), work done above the rate of end test power (W^ETP), total work done (TWD).

IPC failed to elicit an ergogenic effect over any of the performance characteristics of the AOT, which is in direct contrast to the increase in critical power reported by Griffin and colleagues during a 3‐min maximal cycling effort (Griffin et al. 2018). However, a closer inspection of the difference between critical power these authors report (IPC: 241 ± 65 W; SHAM: 234 ± 67 W) would suggest the magnitude to be trivial (*d* < 0.11) (Griffin et al. 2018). Nonetheless, neither Griffin's nor this study observed any effect of IPC on TSI during the exercise task, with both reporting around a 20–25% reduction regardless of condition from the start to the end of the 3 min (Fig. 2). This again may be a function of task demand, as others showing improvements during exercise to failure after IPC consistently demonstrate amplified and accelerated deoxyhemoglobin dynamics (Griffin et al. 2018; Halley et al. 2018). A recent meta‐analysis gives further support, reporting the likelihood of IPC providing an ergogenic benefit in time to failure tasks at nearly 6%, whereas only a 0.5% improvement persists in those incorporating fixed end‐points (Salvador et al. 2016). In many of the fixed‐endpoint tasks (excluding repeat sprints), there have also been incidences of improved oxygen delivery in the absence of any performance (Zinner et al. 2017; Halley et al. 2018; Turnes et al. 2018). Whilst it is well established that IPC induces the production of vasodilatory factors (nitric oxide, adenosine, etc.) (Kimura et al. 2007), the attenuation of oxidative stress and preserved mitochondrial function may play a greater role in improving performance (Semenza 2011). In relation to task demand, tasks to failure may benefit to a greater extent from attenuations in oxidative stress than in fixed‐endpoint tasks. The exception to this might be when additional stressors are imposed during the fixed‐endpoint tasks to induce a comparable oxidative stress, such as exercising in hypoxia (Kjeld et al. 2014; Paradis‐Deschenes et al. 2018). For example, the ergogenic effect of IPC on 5 km cycling time trial (fixed‐end point) performance has been shown to increase under progressively hypoxic conditions (F_I_O_2_ 0.18: +1.1%; F_I_O_2_ 0.15: +1.7%) (Paradis‐Deschenes et al. 2018).

A consistent limitation of IPC research is the inability to truly blind participants to the significant pressure differences between the SHAM and IPC protocols. To account for this in this study, participants were informed that both conditions may influence performance; however, they were naïve to the expected outcome. The efficacy of this control measure was evidenced in the lack of difference between performance responses, consistent MVC and RT reductions (~ 36%, ~ 32%) and the similar magnitude and timing of peak deoxygenation levels (~139 AU, ~35 s) across all IPC and SHAM trials.

## Conclusion

This experiment used a dynamic, 3‐min leg extension protocol to promote muscle oxygenation in order to assess the effect of IPC on exercise‐induced reductions in voluntary activation and sEMG amplitudes. Our findings suggest that IPC does not influence voluntary activation to the muscle during such a task, nor does it affect tissue oxygenation or functional measures of performance. Future research investigating the influence of task demand and exercise severity is warranted to further explore the mechanisms that underpin performance improvements after IPC.

## Conflict of Interest

None declared.
